# The Role of MRI in Prostate Cancer Active Surveillance

**DOI:** 10.1155/2014/203906

**Published:** 2014-11-30

**Authors:** Linda M. Johnson, Peter L. Choyke, William D. Figg, Baris Turkbey

**Affiliations:** ^1^Molecular Pharmacology Section, Medical Oncology Branch, National Cancer Institute, Bethesda, MD 20852, USA; ^2^Molecular Imaging Program, National Cancer Institute, Bethesda, MD 20852, USA; ^3^Clinical Pharmacology Program, Center for Cancer Research, National Cancer Institute, Bethesda, MD 20852, USA

## Abstract

Prostate cancer is the most common cancer diagnosis in American men, excluding skin cancer. The clinical behavior of prostate cancer varies from low-grade, slow growing tumors to high-grade aggressive tumors that may ultimately progress to metastases and cause death. Given the high incidence of men diagnosed with prostate cancer, conservative treatment strategies such as active surveillance are critical in the management of prostate cancer to reduce therapeutic complications of radiation therapy or radical prostatectomy. In this review, we will review the role of multiparametric MRI in the selection and follow-up of patients on active surveillance.

## 1. Introduction

Prostate cancer is the most commonly diagnosed noncutaneous cancer and is the second leading cause of cancer death in American men [1]. In the United States alone, approximately 233,000 men will receive a diagnosis of prostate cancer and 30,000 will die of this disease in 2014 [1]. The clinical behavior of prostate tumors varies from low-grade indolent tumors to aggressive tumors that may progress to metastases and death. While the number of men diagnosed with prostate cancer is increasing, only one in thirty-three men diagnosed with prostate cancer will die of castration resistant metastatic disease [2]. Patients with low-risk disease (characterized by Gleason ≤ 6, PSA < 10, and clinical stage ≤ T2a) have an even more favorable prognosis. Considering these facts, conservative treatment strategies such as active surveillance are critical in the management of prostate cancer to reduce therapeutic complications of radiation therapy or radical prostatectomy. Herein, we will review the role of multiparametric MRI (MP-MRI) in the selection and follow-up of active surveillance patients.

## 2. Selection of Active Surveillance Patients

Active surveillance is a viable option for patients who are candidates for curative treatment but do not require immediate intervention at the time of their diagnosis. Because most low-risk prostate tumors have an indolent course and the slow growth rate allows ample time during follow-up to detect tumors that begin more aggressive while still remaining in a window of definitive curability [3]. The goal of placing patients on active surveillance is to avoid the side effects of radical treatment and offer definitive therapy only if the disease progresses. By offering active surveillance to patients with low risk of local disease progression, patients are able to avoid or postpone adverse side effects of radical prostatectomy or radiation therapy until a later point in time when disease progression warrants radical local treatment. The risk of this approach is that treatment may not be initiated sufficiently early to offer curative treatment in the case of tumor progression.

Published active surveillance protocols vary by institution and rely on PSA levels, digital rectal exam, and TRUS-guided biopsy results. The majority of protocols will include only patients with Gleason 3 + 3 disease; however, a subset of patients with intermediate risk Gleason 3 + 4 disease may also be candidates for active surveillance [4, 5]. The presence of a Gleason 4 pattern indicates more aggressive disease, and the percentage of Gleason 4 in the biopsy specimen is the best predictor of lymph node metastasis [6]. A large volume of Gleason 4 disease warrants definitive treatment in most patients. However, patients with a very small volume of Gleason 4 and PSA < 10 may have been shown to have a disease comparable to Gleason 3 + 3 [7–9].

A negative prostate MP-MRI has been shown to have greater than a 95% negative predictive value for clinically significant cancer [10, 11]. Thus a patient with a negative MRI and low-risk disease may be advised to pursue active surveillance. There have been several studies showing that patients with visible lesions on MP-MRI have an increased overall risk of cancer progression. Fradet et al. found that a lesion on MP-MRI tripled the risk of overall cancer progression [12]. Park et al. showed that patients without a discrete tumor on MP-MRI are more suitable for active surveillance than those with a visible tumor on MP-MRI [13]. Another study of 60 patients with PSA < 10 ng/mL and no more than 3 cores of Gleason 3 + 3 examined patients at the outset of active surveillance and found that men with a negative MRI had only a 3.5% chance of reclassification to intermediate or high risk disease on confirmatory biopsy of cores targeted to the MRI visible lesion [14]. Turkbey et al. examined 133 patients and found that the sensitivity and overall accuracy for predicting active surveillance candidates were 93% and 92%, respectively, suggesting that MP-MRI image evaluation improves the identification of patients eligible for active surveillance when used in conjunction with clinical-pathologic criteria [15]. MRI-guided biopsies give a more accurate picture of the disease burden, with little upgrading between MRI-TRUS-guided biopsy and radical prostatectomy [16].

## 3. MP-MRI for Prostate Cancer Detection

MP-MRI is the combination of multiple MRI sequences to give both anatomical and functional information about suspicious lesions, usually consisting of T1-weighted MRI (T1W MRI), T2-weighted MRI (T2W MRI), diffusion-weighted MRI (DW MRI), dynamic contrast-enhanced MRI (DCE MRI), and MR spectroscopy (MRSI). The combination of imaging findings from all of these parameters improves the accuracy of MP-MRI and allows a tumor suspicion level to be assigned. Considering the unreliability of relying on PSA testing for prostate cancer diagnosis, the ability to visualize the tumor and assess tumor volume is becoming critical. Accurate imaging of prostate lesions and the ability to use these images for imaged guided biopsies are a major advancement in assessment of patients eligible for active surveillance and for continued follow-up in the surveillance period.

Either pelvic or endorectal coils (ERC) may be used when performing a MP-MRI scan of the prostate to obtain higher signals. ERC improves resolution of the images but increases patient discomfort and requires a trained team of a technician and radiologist to perform the procedure. For these reasons MP-MRI with ERC is not routinely used for diagnostic scans. Either a 3 Tesla or 1.5 Tesla magnet can be used for MP-MRI of the prostate; however, 3 Tesla magnets are advantageous because they improve signal-to-noise ratios and require shorter image acquisition times than 1.5 Tesla magnets.

T1W MRI provides anatomical identification of areas of hemorrhage within the prostate since blood displays a high signal intensity against a homogenous low signal background on T1W images [17]. This is useful in the identification of postbiopsy hemorrhage, which can interfere with tumor detection since areas of inflammation appear similar to tumor on T2W MRI. Biopsy procedures can also cause capsular irregularity which may mimic extracapsular extension. For these reasons, many radiologists recommend waiting at least 6–8 weeks after biopsy procedures before performing a prostate MRI, and some clinics recommend waiting even longer, up to 10–12 weeks.

T2W MRI is the foundation of MP-MRI and allows visualization of the prostate anatomy. Due to its high water content, the peripheral zone normally displays high signal intensity. By contrast, tumors display low signal intensity in the peripheral zone, allowing identification of suspicious lesions [17]. However, low signal areas on T2W MRI in the peripheral zone may also be due to many benign conditions such as inflammation and postbiopsy scarring or hemorrhage [17]. Guidelines published by the ESUR state that T2 sequences should include the prostate, seminal vesicles, and external sphincter with ≤3 mm section thickness and in-plane resolution of 0.7 mm or better [18].

DW MRI measures the diffusion of water within the extracellular space, specifically looking at the water proton diffusion using the apparent diffusion coefficient (ADC) [19, 20]. Because cancer cells are more tightly packed than benign cells, diffusion of water is restricted in cancerous lesions, resulting in a decreased signal on the ADC map, which is constructed over multiple *b* values [21, 22].

DCE MRI uses a series of T1-weighted images rapidly obtained after injection of an intravenous contrast agent to measure the vascularity of the tissue. The ESUR suggests a bolus injection at 3 mL/sec with a standard dose of contrast medium and recommends a minimum slice thickness of 4 mm [18]. Tumors have increased vascularity due to neoangiogenesis related to tumor aggressiveness and therefore they take up the contrast agent more rapidly than normal tissue [23]. This contrast washes out of tumor regions quickly, leading to a steep wash in-wash out enhancement curve. DCE MRI has a high sensitivity, which is useful in the initial diagnostic evaluation of suspicious lesions, and is routinely used to detect lesions and monitor therapeutic responses to treatment [24, 25].

MRSI utilizes the different metabolites in tumor tissue versus benign tissue; cancerous cells contain more choline due to increased cell turnover, leading to an increased choline : citrate ratio [26–28]. This technique requires the use of endorectal coil and has not been shown to consistently improve diagnostic performance to identify suspicion lesions and guide biopsies. Furthermore MRSI sequences require a long image acquisition time and require trained radiologists for proper shimming and data interpretation, adding costs to the MP-MRI studies. For these reasons, MRSI is less commonly performed than other MP-MRI sequences in prostate MRI studies.

## 4. The Use of MP-MRI in Prostate Biopsy

Prostate biopsy is considered a safe procedure, with <0.1% risk of sepsis and other mild side effects such as hematospermia, hematuria > 1 day, rectal bleeding, and prostatitis in 37.4%, 14.5%, 2%, and 1% of patients, respectively [29]. Although hematospermia is usually not clinically significant, patients should be advised that it may last several weeks to avoid unnecessary anxiety. Because fecal matter may be introduced into the prostate and cause infection during a transrectal biopsy procedure, the use of prophylactic antibiotics such as fluoroquinolones is recommended [30].

Indications for prostate biopsy include a palpable nodule on digital rectal examination, clinical symptoms, suspicion for prostate cancer, high PSA value, or high PSA velocity (rate of change in PSA levels). Prostate cancer remains the only solid malignancy in which biopsy procedures are not directed at particular lesions. The current standard of care for prostate biopsy procedures is to perform a 12–14-core random transrectal ultrasound- (TRUS-) guided biopsy, during which the urologist attempts to systematically sample tissue from the apex, mid, and base regions of the prostate. Traditional methods of prostate cancer detection, including TRUS-guided biopsy in conjunction with serum PSA testing and digital rectal exams, have low sensitivity with only a 24–44% success rate of cancer detection, which may lead to underdiagnosis of large volume clinically significant tumors and overdetection of low-grade tumors, particularly in the anterior prostate gland which is a difficult area to biopsy using the TRUS-guided technique [31–33]. While MRI can be used directly to guide biopsy procedures, MRI is time consuming and costly and requires the patient to remain in the MR gantry for the entire procedure [34]. A more rational solution is to use MRI-TRUS fusion guidance in which image registration between the MRI and ultrasound is achieved by automated segmentation with the opportunity for manual adjustment during the procedure to compensate for movement of the prostate gland.

The addition of MP-MRI to this biopsy strategy or, in select patients, using MP-MRI as a substitute for a repeat biopsy improves prostate cancer detection [10, 35–37]. By performing MP-MRI before TRUS-guided biopsy, lesions identified on MP-MRI can be targeted for biopsy rather than relying solely on the systematic random sampling of the posterior prostate. Targeting biopsies to abnormal regions of the prostate as identified on MP-MRI detects clinically significant prostate cancer in an equivalent or higher percentage of patients and results in lower diagnosis rates of clinically insignificant tumors [38]. One study of 1448 patients with suspicion of prostate cancer underwent either targeted or systematic biopsies; the cancer detection rate was higher in the targeted biopsy group with a positive predictive value (PPV) of MR findings of 70.1%–90.1% [39]. Siddiqui et al. showed in a study of 582 patients that targeted biopsy detected 67% more Gleason ≥ 4 + 3 tumors than 12-core biopsy alone and missed 36% of Gleason ≤ 3 + 4 tumors, thus minimizing the detection of clinically insignificant disease [40].

MR-TRUS fusion allows the MRI to direct biopsy needles under TRUS guidance, thus combining MRI's high sensitivity for identifying suspicious lesions with the practicality of TRUS biopsy procedures. MRI-US fusion targeted prostate biopsies help to avoid detection of clinically insignificant tumors while allowing diagnosis of serious tumors difficult to detect by conventional biopsy techniques, such as tumors in the anterior, midline, and apex of the prostate, which are often undersampled by systematic biopsy procedures that only sample the lateral peripheral zone [41]. Therefore MRI-TRUS fusion guided biopsies allow the urologist to take advantage of the sensitivity of MRI to improve prostate cancer diagnosis in an outpatient office-based procedure.

MR-TRUS fusion biopsy procedures can be performed in the urologist office setting and allow targeting of suspicious lesions seen on MP-MRI for biopsy under real-time US. Biopsy cores obtained from MR-TRUS fusion have twice the detection rate of random TRUS biopsy and detect higher risk cancers, with the targeted biopsies detecting 67% more Gleason ≥ 3 + 4 tumors but missing 36% of the Gleason ≤ 3 + 4 tumors [42]. Targeted biopsies therefore selectively sample suspicious lesions and preferentially biopsy higher grade tumors which are more likely to require therapeutic intervention. MR-TRUS fusion biopsy also improves cancer detection rates in patients with enlarged prostates and patients with a history of multiple prior negative biopsies, in whom detection rates are lower via conventional random biopsy techniques.

## 5. Serial MP-MRI Scans

When following patients on active surveillance, serial MP-MRI scans are critical during follow-up to detect progression of visible lesions over time. By assessing tumor characteristics over time in a noninvasive manor, repeat MP-MRI scans build up a radiological phenotype including tumor size and functional tumor characteristics such as vascularity and cell density. MP-MRI offers the opportunity to assess tumor volume, aggressiveness, and containment within the prostate and can be repeated over time in patients on active surveillance [43]. Furthermore, the accuracy of MP-MRI improves with increasing tumor grade and volume [44]. Because MP-MRI shows only more significant tumors, repeat imaging in patients on active surveillance helps to identify low-risk lesions which may have progressed to intermediate or high-grade lesions according to functional MP-MRI characteristics. Patients with a visible lesion on MP-MRI are more likely to show radiological progression than patients with no visible lesions [45]. Rais-Bahrami et al. examined 153 patients who underwent MP-MRI and subsequent MR-TRUS fusion guided biopsy and found no significant change in lesion size in patients with ≤7 mm index lesions after a minimum two-year interval between their initial and most recent MP-MRI [46]. Stevens show that, in a series of 108 men, 45% of those with a visible lesion showed radiological progression compared with 17% of those with no visible lesion at baseline [45]. The natural history of MP-MRI lesions in patients on active surveillance is not fully explored, and standardized criteria are needed to define radiological progression for patients on active surveillance that incorporates tumor volume and functional parameters such as diffusion-weighted and contrast-enhanced images [47].

## 6. MP-MRI as Replacement for Repeat Biopsy

In patients with a prior negative biopsy result, the effect of a positive biopsy result on clinical management must be considered. The presence of lesions visible on MP-MRI, a family history of prostate cancer, increasing PSA levels, palpable lesions, or clinical symptoms should warrant further work-up, particularly in younger and healthier patients, in whom there is a greater survival benefit by pursuing prostate cancer treatment. In patients who have undergone at least one negative standardized TRUS biopsy, cancer detection rates are 38%–59% [48]. If the MP-MRI finding is stable over time since the prior MP-MRI and the previous biopsy showed low-risk disease, it is reasonable to forgo the biopsy and allow the MRI findings to substitute for the biopsy procedure.

## 7. MP-MRI in Prostate Cancer Staging

MP-MRI can help assess the risk of prostate tumors based on the MP-MRI appearance of the lesions. MP-MRI is recommended when a patient is considered for active surveillance because it allows detection of poor prognostic features such as large volume or high-grade tumors, particularly in the anterior prostate. A study of 800 patients by Yerram et al. who underwent 3T MP-MRI of the prostate demonstrated that patients with low suspicion lesions on MP-MRI were more likely to have negative biopsies or low-grade tumors, suggesting that patients with low suspicion lesions on MP-MRI have a sufficiently small risk of clinically significant disease to justify pursuing active surveillance [49].

## 8. Conclusion

Active surveillance is gaining popularity as a cancer management option for patients with low-risk localized prostate cancer since it reduces the risk of overtreatment of patients with clinically insignificant disease [50, 51]. In addition, with other clinical variables such as age, PSAD, and family history, MP-MRI can help identify patients with prostate cancer who are eligible for active surveillance as an initial therapeutic management strategy. MP-MRI before biopsy is useful for lesion detection and permits MP-MRI-targeted TRUS fusion biopsy procedures, which preferentially samples suspicious lesions and allows detection of areas of the prostate such as the anterior prostate which are hard to be biopsied using traditional random biopsy techniques. The risk of clinically significant disease in patients with a negative MP-MRI may be sufficiently low to consider deferring definitive treatment for active surveillance and further studies are warranted.
